# Learning by Doing…Errors! Comment on Gil-Lacruz et al. Learning by Doing and Training Satisfaction: An Evaluation by Health Care Professionals. *Int. J. Environ. Res. Public Health* 2019, *16,* 1397

**DOI:** 10.3390/ijerph17010051

**Published:** 2019-12-19

**Authors:** Michela Cortini

**Affiliations:** Dipartimento di Scienze Psicologiche, della Salute e del Territorio, Università G. d’Annunzio of Chieti-Pescara, 66100 Chieti, Italy; cortini@unich.it

In their recent paper [1], Gil-Lacruz and colleagues provided a very interesting study on professional training, seen as a positive measure for improving the quality of the system and a strategy for the prevention of stress and an improved working environment. In particular, they offer an interesting view of a specific kind of training; that is, learning by doing. While this paper contributes to providing an overview of workplace learning by doing in the health sector, stressing all the possible outcomes that are identified and explained, some—a few, to be honest—issues could be further addressed.

First of all, the authors, according to their research aims, frame learning as being, predominantly, an individual-based phenomenon: the process of learning seems to be something that happens between a singular employee and her/his organization. Actually, a growing body of literature refers to learning organizations [2,3,4] and the learning climate, stressing that there are organizational conditions that facilitate learning processes, especially in the health sector [5,6].

This is particularly true for a specific way of learning by doing, which occurs when we make mistakes. In fact, if, in more traditional ways of learning by doing, workers learn by themselves, in error management training, there is the possibility to learn by one’s own errors but also from those of others.

Last but not least, even if the literature stresses the idea of life-long-learning, something explicitly shared by Gil-Lacruz and collegues—meaning that we have to learn on a continuous basis—it would be important to distinguish among early-career, middle-career and late-career stages; especially for the early-career and apprenticeship period, the possibility of making errors and learning from them should be supported by specific training programmes [7,8].

To conclude, health policies related to health care professionals could consider error management programs as a key resource to develop learning and improvement processes in their employees and organizations.
